# Spitz nevus in a patient with oculocutaneous albinism: dermoscopic and histopathologic correlation^؆^

**DOI:** 10.1016/j.abd.2026.501360

**Published:** 2026-05-14

**Authors:** Julia Aires Thomaz Maya, Priscila Ishioka, Leonardo Romaniello Gama de Oliveira, Carolina Reato Marçon

**Affiliations:** a Dermatology Clinic, Hospital da Santa Casa de São Paulo, Santa Casa de Misericórdia de São Paulo, São Paulo, SP, Brazil; b Department of Pathology, Hospital da Santa Casa de São Paulo, Santa Casa de Misericórdia de São Paulo, São Paulo, SP, Brazil; c Faculty of Medical Sciences, Santa Casa de Misericórdia de São Paulo, São Paulo, SP, Brazil

Dear Editor,

In oculocutaneous albinism (OCA), an autosomal recessive disorder characterized by total or partial absence of melanin production, melanocytic lesions pose a diagnostic challenge due to their atypical dermoscopic presentation.^1^ The present report describes a patient with OCA followed by digital mapping, in whom a Spitz nevus (SN) was identified and confirmed by histopathologic examination.

A 25-year-old female patient with OCA, with no family history of albinism or other skin diseases, underwent digital dermoscopic follow-up at a tertiary dermatology center due to multiple atypical nevi. During the dermoscopic follow-up, a 0.4 cm light brown papule was identified on the left upper limb. Dermoscopy revealed globular structures with a yellowish appearance and uniform distribution throughout the lesion (Fig. 1) and fine punctate vessels, distributed globally and symmetrically (Fig. 2). Given the atypical appearance of the lesion, excision was chosen. The lesion area had been examined approximately one year prior without any lesion being detected.

**Figure 1:**
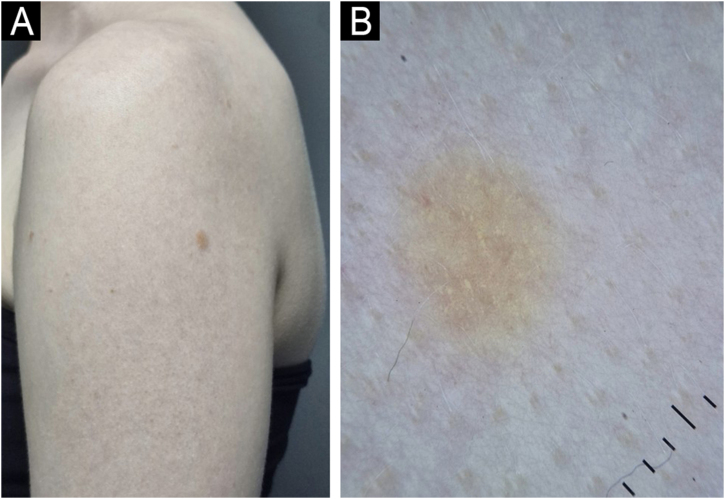
(A) Patient with oculocutaneous albinism presenting with a 0.4 cm non-pigmented nevus on the left upper limb. (B) Dermoscopy with polarized light reveals yellowish structures in the central region.

**Figure 2:**
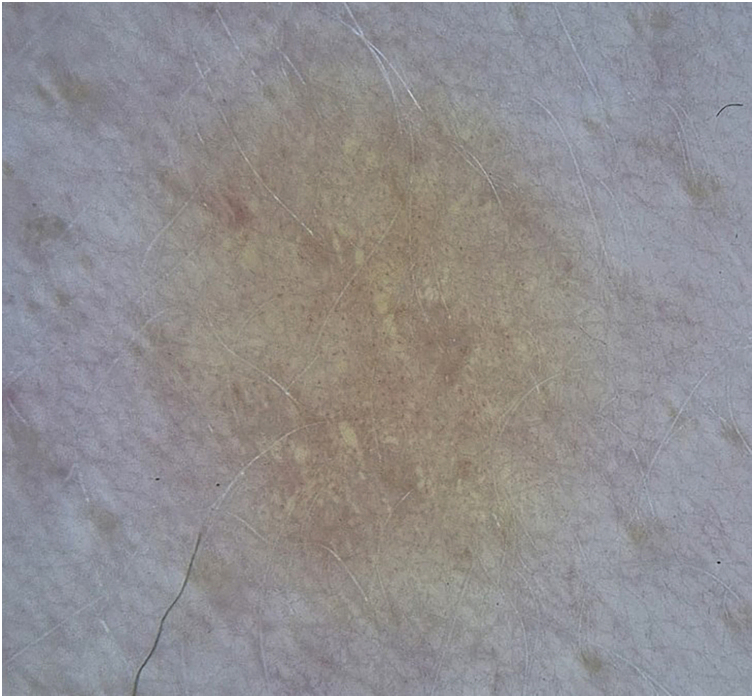
Dermoscopic image of the lesion obtained with polarized light showing millimeter-sized punctate vessels, distributed globally and symmetrically throughout the lesion at higher dermoscopic magnification.

The evaluation of the histological sections revealed a well-defined, compound melanocytic proliferation, formed by large and varied nests of epithelioid and fusiform melanocytes. The dermal component was restricted to the superficial reticular dermis. Some cells showed ample cytoplasm, sometimes granular, with discrete cytological atypia. Junctional nests showed separation gaps in relation to the adjacent epidermis, in addition to eosinophilic globular structures compatible with Kamino bodies. Subtle acanthosis was observed, without mitotic activity or pagetoid dissemination of melanocytes. There was no melanocytic pigmentation (Fig. 3). Immunohistochemistry showed preserved p16 expression, negative BRAF test, and positivity for Melan-A (Fig. 4). The test was reviewed by two experienced dermatopathologists, who confirmed the diagnosis of Spitz nevus.

**Figure 3:**
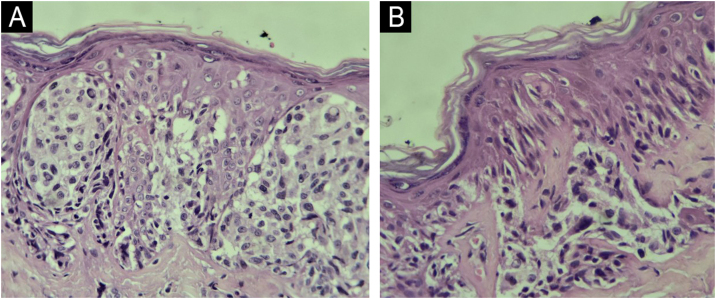
Patient's histopathologic examination. (A) Large nests of epithelioid and spindle cells with ample cytoplasm. Slight acanthosis can be observed. (Hematoxylin & eosin, ×10). (B) Irregular nests of epithelioid and spindle cells, presence of Kamino bodies (Hematoxylin & eosin, ×10).

**Figure 4:**
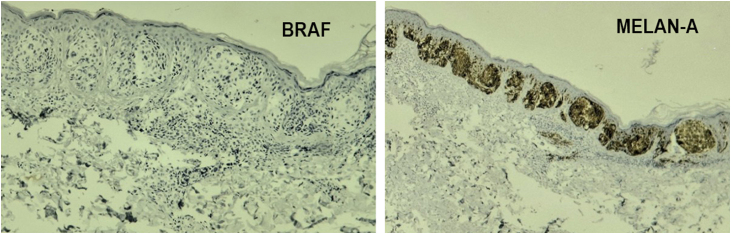
Immunohistochemistry examination of the patient: left showing negative BRAF staining in cells of interest (BRAF ×10); right showing positive Melan A staining in cells of interest (Melan A ×10).

Spitz nevus is a melanocytic neoplasm consisting of epithelioid and/or spindle cells. Clinically, it presents as a solitary, well-defined papule or nodule, usually <1 cm, with rapid initial growth and predominance on the limbs of young adults. The classic dermoscopic presentation involves a punctate vascular pattern in about 50% of cases, with regularly distributed monomorphic vessels on a homogeneous pink background. Other, less common patterns include reticular or homogeneous depigmentation.^2^

Histologically, it is characterized by symmetry, well-defined delimitation, presence of epithelioid/fusiform melanocytes, progressive dermal maturation, mild cytological atypia, Kamino bodies, occasional mitoses, and discrete lymphocytic inflammatory infiltrate.^3^, ^4^ The preserved p16 expression observed in the patient reinforces the lesion's benignity, since its loss is associated with malignant Spitzoid neoplasms. Melan-A, in turn, confirmed the melanocytic nature.^5^

From a dermoscopic point of view, the yellowish globules observed may correspond to the melanocytic nests identified histologically, whose absence of pigment in the context of OCA results in a yellowish hue instead of the homogeneous pink background coloration usually observed.

Regarding molecular biology, activating mutations in BRAF and NRAS are known to be rare in Spitz nevi, although age may influence their occurrence. Fusions involving BRAF are described in about 5% of epithelioid lesions, and a small percentage may evolve into melanoma.^6^, ^7^ The negative result for BRAF in this case is consistent with the expected profile for Spitz nevi, which usually show gene fusions that are not detectable by this method.^8^

Despite the extensive description of Spitz nevi in ​​the literature, there is still a scarcity of data on their dermoscopic presentation in patients with oculocutaneous albinism, which reinforces the relevance of this report. In particular, the observation of yellowish structures under polarized light is highlighted, which, in hypopigmented lesions, may correspond to clusters of melanocytic cells, a finding of potential diagnostic value. During the literature review, the authors did not find a dermoscopic description of Spitz nevus in patients with oculocutaneous albinism.

## ORCID ID

Priscila Ishioka: 0000-0001-9686-1902

Leonardo Romaniello Gama de Oliveira: 0000-0003-0986-1264

Carolina Reato Marçon: 0000-0001-8261-3166

## Research data availability

Does not apply.

## Financial support

None declared.

## Authors' contributions

Julia Aires Thomaz Maya: Collection of data, or analysis and interpretation of data; drafting and editing of the manuscript or critical review of important intellectual content; acquisition, analysis, and interpretation of data; critical review of the literature; approval of the final version of the manuscript.

Priscila Ishioka: Collection of data, or analysis and interpretation of data; drafting and editing of the manuscript or critical review of important intellectual content; acquisition, analysis, and interpretation of data; critical review of the literature; approval of the final version of the manuscript.

Leonardo Romaniello Gama de Oliveira: collection of data, or analysis and interpretation of data; drafting and editing of the manuscript or critical review of important intellectual content; acquisition, analysis, and interpretation of data; critical review of the literature; approval of the final version of the manuscript.

Carolina Reato Marçon: Design and planning of the study; collection of data, or analysis and interpretation of data; statistical analysis; drafting and editing of the manuscript or critical review of important intellectual content; acquisition, analysis, and interpretation of data; effective participation in research orientation; intellectual participation in the propaedeutic and/or therapeutic conduct of the studied cases; critical review of the literature; approval of the final version of the manuscript.

## Conflicts of interest

None declared.

## References

[1] Bakos R.M., Argenziano G., Zalaudek I., Masiero N.C., Zoratto G., Cartell A. (2009). Dermatoscopy of pigmented melanocytic nevi in patients with oculocutaneous albinism. J Am Acad Dermatol..

[2] Casso E.M., Grin-Jorgensen C., Grant-Kels J.M. (1992). Spitz nevi. J Am Acad Dermatol..

[3] Chatzopoulos K., Syrnioti A., Linos K. (2024). Spitz melanocytic tumors: a fascinating 75-year journey. Genes (Basel)..

[4] Gerami P., Chen A., Sharma N., Patel P., Hagstrom M., Kancherla P. (2024). BRAF mutated and morphologically Spitzoid Tumors, a subgroup of Melanocytic Neoplasms difficult to distinguish from true Spitz Neoplasms. Am J Surg Pathol..

[5] Garrido-Ruiz M.C., Requena L., Ortiz P., Pérez-Gómez B., Alonso S.R., Rodríguez Peralto J.L. (2010). The immunohistochemical profile of Spitz nevi and conventional (non-Spitzoid) melanomas: a baseline study. Mod Pathol..

[6] McAfee J.L., Scarborough R., Jia X.S., Azzato E.M., Astbury C., Ronen S. (2023). Combined utility of p16 and BRAF V600E in the evaluation of spitzoid tumors: superiority to PRAME and correlation with FISH. J Cutan Pathol..

[7] Ferrara G., Gianotti R., Cavicchini S., Salviato T., Zalaudek I., Argenziano G. (2013). Spitz tumor, and spitzoid melanoma: a comprehensive clinicopathologic overview. Dermatol Clin..

[8] Sainz-Gaspar L., Sánchez-Bernal J., Noguera-Morel L., Hernández-Martín A., Colmenero I., Torrelo A. (2020). Spitz Nevus and other spitzoid tumors in children - part 1: clinical, histopathologic, and immunohistochemical features. Actas Dermosifiliogr (Engl Ed)..

